# Advances in Condition Monitoring of Railway Infrastructure

**DOI:** 10.3390/s24030830

**Published:** 2024-01-26

**Authors:** Araliya Mosleh, Diogo Ribeiro, Abdollah Malekjafarian, Maria D. Martínez-Rodrigo

**Affiliations:** 1 CONSTRUCT—LESE, Faculty of Engineering, University of Porto, 4200-465 Porto, Portugal; 2 CONSTRUCT—LESE, School of Engineering, Polytechnic of Porto, 4249-015 Porto, Portugal; drr@isep.ipp.pt; 3 Structural Dynamics and Assessment Laboratory, School of Civil Engineering, University College Dublin, Belfield, D04V1W8 Dublin, Ireland; abdollah.malekjafarian@ucd.ie; 4 Mechanical Engineering and Construction Department, Universitat Jaume I, 12006 Castellón, Spain; mrodrigo@uji.es

In recent years, there has been a notable surge in investments directed towards developing new railway lines and revitalising existing ones, reflecting a global commitment to enhance transportation infrastructure. This wave of investment is essential in meeting the growing demands of modern societies for efficient and sustainable transportation options. These efforts encompass numerous critical infrastructures within the railway network, ranging from bridges and tunnels to tracks and signaling systems. Therefore, it is imperative to ensure the operational integrity and safety of these infrastructures throughout their life cycle, safeguarding against potential hazards and ensuring uninterrupted service [1,2].

This imperative has catalyzed significant advancements in the field of structural condition monitoring for railway infrastructures [3,4]. In addition, recent scientific and technological breakthroughs have transformed the way these infrastructures are monitored and maintained.

Moreover, integrating artificial intelligence (AI) and machine learning algorithms has been decisive in enhancing the accuracy and efficiency of structural condition assessment [5,6,7]. These technologies can process large amounts of data, identifying early anomalies and trends that may indicate forthcoming critical situations. Additionally, using unmanned aerial vehicles (UAVs) equipped with advanced imaging and sensing capabilities has paved the way for cost-effective and comprehensive inspections of hard-to-reach or hazardous areas, thus further supporting the capabilities of railway infrastructure monitoring.

The significance of these advancements in ensuring the longevity and safety of railway infrastructures cannot be overstated. They not only contribute to the overall efficiency and reliability of the transportation network but also play a crucial role in minimizing the environmental footprint associated with maintenance activities. The synergistic combination of strategic investments and innovative technological applications underscores a concerted effort toward building a resilient and sustainable railway infrastructure network capable of meeting future demands. Despite advancements in the railway industry in recent years, there remains a noticeable gap in the ability of science and technology to instigate transformative innovations in the railway industry at its core.

This Editorial refers to the Special Issue “Advances in Condition Monitoring of Railway Infrastructures”, which serves as a compilation of the most recent research achievements in the scope of advanced planning, design, construction, monitoring, maintenance, and management of railway infrastructures. A total of 20 manuscripts were submitted for evaluation in the context of the Special Issue, with each manuscript undergoing a comprehensive and strict review process. Subsequently, 10 papers have been accepted for publication, and their respective contributions are detailed in the List of Contributions.

As indicated above, the contributions span diverse geographical regions, encompassing specific country cases such as China, Spain, Poland, Ireland, France, Portugal, Netherlands, and Germany. The topics covered include AI (1, 3, 4, 6), track monitoring approaches (1 and 4), wayside wheel defect detection (6), freight wagon digitalization (5), digital twins (7), Structural Health Monitoring (SHM) (9), and tunnels (10). Contributions 4, 6, and 9 entail numerical studies, while contributions 1, 2, 3, 5, 7, and 8 involve experimental field tests.

Contribution 1 introduces a rail surface defect detection model, denoted as FS-RSDD, designed for the rail surface condition monitoring. Notably, it addresses the prevalent challenge of limited defect samples encountered by prior detection models. The proposed model leverages a pre-trained framework to extract features from both normal and defective rail specimens. Subsequently, an unsupervised learning approach is employed to discern feature distributions and establish a feature prototype memory bank. Employing prototype learning strategies, FS-RSDD computes the likelihood of a test sample being associated with a defect at each pixel, guided by the information stored in the prototype memory bank. This methodology mitigates the constraints faced by deep learning algorithms based on supervised learning paradigms, which often deal with inadequate training samples and reduced reliability in validation. FS-RSDD attains noteworthy precision in defect detection and localization, even when trained with a restricted number of defective samples.

Contribution 2 explores the condition monitoring of an overhead contact line (OCL) by creating a monitoring system tailored for a pantograph installed on multiple electrical units. Kinematic and dynamic modeling of the pantograph is undertaken to support the development of the monitoring system. This modeling is validated through meticulous test-rig experiments, after which the proposed methodology is subjected to comprehensive field tests serving a dual purpose: firstly, to prove the efficiency of the monitoring system using benchmark measurements obtained from the tCat^®^ trolley, and secondly, to evaluate the reproducibility of measurements under realistic operation scenarios.

Contribution 3 addresses object detection using computer vision in scenarios characterized by limited data by training YOLOv5 and MobileNet frameworks. A dataset comprising 120 observations was demonstrated to be adequate for achieving high accuracy in the object detection task specific to railway infrastructure. Additionally, a novel approach for the extraction of background images from railway imagery was introduced. To validate this method, the performance of YOLOv5 and MobileNet was evaluated on small datasets, both with and without background extraction. The experimental outcomes indicate that the application of background extraction reduces the sufficient data volume to 90.

In Contribution 4, a novel approach for monitoring railway track conditions is presented, based on acceleration responses obtained from an operational train to detect alterations in the stiffness of underlying track sub-layers. An artificial neural network (ANN) algorithm is formulated, operating on the energy content of the train’s acceleration responses. A computational model of a half-car train interacting with a track profile is employed to simulate the vertical acceleration of the train. The induced damage is represented by reduced soil stiffness within the sub-ballast layer, representative of voided sleepers. Furthermore, a sensitivity analysis is conducted to evaluate the influence of signal noise, slice sizes, and the presence of multiple damaged locations on the performance of the damage index.

Contribution 5 presents ongoing progress in the digitalization of freight wagons, encompassing the delineation, fabrication, and on-site trials conducted on a commercial rail line in Sweden. A diverse array of components and systems were installed in a freight wagon, envisaging the completion of an intelligent freight wagon. The digitalization effort encompasses the seamless integration of sensors designed to provide various functions, including but not limited to train composition analysis, train integrity assessment, asset monitoring, and continuous wagon positioning. These strides herald the potential for real-time data analysis, anomaly detection, and the implementation of proactive maintenance strategies, envisaging the operational efficiency and safety of freight transportation.

Contribution 6 assesses and compares the effectiveness of four distinct feature extraction methodologies, specifically, the auto-regressive (AR) method, auto-regressive exogenous (ARX) method, principal component analysis (PCA), and continuous wavelet transform (CWT), in their capacity to autonomously differentiate between a defective wheel and a healthy one. The reference measurement employed in this investigation is the rail acceleration during the transit of freight vehicles. The study encompasses four sequential steps: (i) feature extraction; (ii) feature normalization; (iii) data fusion; and (iv) damage detection. The findings of this study underscore that the AR and ARX extraction methods exhibit superior efficiency in wheel flat damage detection compared to CWT and PCA techniques.

Contribution 7 investigates the applicability of three deep-learning-based models, namely, PointNet++, SuperPoint Graph, and Point Transformer, for the semantic segmentation of point clouds within the context of a practical, real-world scenario. The study centers on a specific use case of catenary arches within the Dutch railway system, conducted in collaboration with Strukton Rail, a prominent contractor for rail projects. A distinctive, complex, high-resolution, and annotated dataset is presented for the evaluation of point cloud segmentation models in railway environments. Comprising 14 individually labeled classes, this dataset represents the first of its kind to be openly accessible. The modified PointNet++ model emerges as the most effective, achieving a mean class Intersection over Union (IoU) of 71% for the semantic segmentation task.

Contribution 8 introduced an ensemble learning approach combining Improved MultiScale Retinex with Color Restoration (IMSRCR) and You Only Look Once (YOLO) based on acquired tunnel image data for the detection of corroded bolts in the lining. The features of the lining images are enhanced and strengthened by IMSRCR, mitigating the adverse effects of a dark environment in contrast to the existing MSRCR. Additionally, models with varying parameters, exhibiting diverse performance characteristics, are integrated using the ensemble learning method, resulting in a substantial improvement in accuracy. Sufficient comparisons based on a dataset collected from the tunnel are conducted to prove the superiority of the proposed algorithm.

Contribution 9 proposes a methodology for axle detection using accelerometers placed arbitrarily on a bridge structure. The model is implemented as a Fully Convolutional Network suitable for processing signals represented in the Continuous Wavelet Transforms format. This allows passages of any length to be processed in a single step with maximum efficiency while using multiple scales in a single evaluation. Consequently, the proposed method can effectively use acceleration signals from any location on the bridge structure, functioning as Virtual Axle Detectors (VADs) without constraint to specific bridge structural types. The efficiency of the proposed method is tested through the analysis of 3787 passages recorded on a steel railway bridge. The results derived from the measurement data indicate that the proposed model successfully detects 95% of the axles.

Contribution 10 seeks to comprehend the disparities in the impact of expressway bridges and subgrades on the near-surface blown sand environment. It examines wind speed and profile variations, wind flow-field characteristics, and sand transport rates around bridges and subgrades. The goal is to offer a scientific foundation for choosing expressway route forms in sandy regions. The study employs wind tunnel tests with models of a highway bridge and subgrade, comparing the environmental effects of wind-blown sand on both structures. The findings hold theoretical and practical importance for guiding expressway route selection in sandy areas.

The Guest Editors are pleased with the conclusive outcomes of the published papers in this Special Issue, anticipating their utility for researchers, engineers, designers, and other professionals engaged in diverse thematic aspects of advanced analytical and numerical simulation approaches, as well as experimental studies, applied to railway infrastructures. The Guest Editors extend their appreciation to all authors and reviewers for their crucial contributions and for the dissemination of scientific findings. Lastly, gratitude is extended to the Editorial Board of *Sensors* for their patience, support, and exceptional contributions.
